# Association between myopia and peripapillary hyperreflective ovoid mass-like structures in children

**DOI:** 10.1038/s41598-020-58829-3

**Published:** 2020-02-10

**Authors:** In Jeong Lyu, Kyung-Ah Park, Sei Yeul Oh

**Affiliations:** 1 Department of Ophthalmology, Samsung Medical Center, Sungkyunkwan University School of Medicine, Seoul, Korea; 2 Department of Ophthalmology, Korea Cancer Center Hospital, Korea Institute of Radiological and Medical Sciences, Seoul, Republic of Korea

**Keywords:** Optic nerve diseases, Risk factors

## Abstract

We investigated the characteristics of children with peripapillary hyperreflective ovoid mass-like structures (PHOMS) and evaluated the associated risk factors. This cross-sectional study included 132 eyes of 66 children with PHOMS and 92 eyes of 46 children without PHOMS (controls) who were assessed by disc enhanced-depth image spectral-domain (SD) optical coherence tomography (OCT). Univariable and multivariable logistic analyses were performed to evaluate risk factors associated with presence of PHOMS. Among the 66 children with PHOMS, 53 (80.3%) had bilateral and 13 (19.7%) had unilateral PHOMS. The mean age of the PHOMS group was 11.7 ± 2.6 years, and that of the control group was 11.4 ± 3.1 years. The mean spherical equivalent (SE) as determined by cycloplegic refraction was −3.13 ± 1.87 diopters (D) in the PHOMS group and −0.95 ± 2.65 D in the control group. Additionally, mean astigmatism was 0.67 ± 0.89 D and 0.88 ± 1.02 D in the PHOMS group and the control group, respectively. Mean disc size was 1,735 ± 153 µm in the PHOMS group and 1,741 ± 190 µm in the control group, while mean optic nerve head (ONH) tilt angle was 9.84 ± 5.38 degrees in the PHOMS group and 3.71 ± 4.41 degrees in the control group. SE and ONH tilt angle were significantly associated with PHOMS according to both univariable [odds ratio (OR): 1.59; *p* < 0.001 and OR: 1.35; *p* < 0.001, respectively] and multivariable (OR: 1.71; *p* = 0.001 and OR: 1.29; *p* = 0.001, respectively) logistic regression analyses. There was a significant correlation between SE and ONH tilt (*r* = −0.46; *p* < 0.001). In conclusion, PHOMS is associated with myopic shift in children, and optic disc tilt may be a mediator between myopia and PHOMS.

## Introduction

The presence of peripapillary hyperreflective ovoid mass-like structures (PHOMS) has arisen as an independent diagnosis recently^1^. In previous studies, PHOMS have been diagnosed as buried optic disc drusen (ODD) or type 2 ODD^2–6^, diseases of the optic nerve head (ONH) with acellular hyaline depositions^7–9^. However, in 2018, the Optic Disc Drusen Studies (ODDS) Consortium defined ODD as hyporeflective structures with full or partial hyper-reflective margin on optical coherence tomography (OCT) and proposed the terminology of PHOMS for the specific finding of hyperreflective mass-like lesions in the peripapillary area^1^. The pathogenesis of PHOMS is suspected as herniation of distended axons into the peripapillary retina^1,10^. The ODDS Consortium reported a histopathologic finding presenting as lateral bulges of the retinal nerve fibers in a patient with papilledema, similar to PHOMS on OCT^1^.

PHOMS can be easily misdiagnosed as papilledema since it presents as an elevated and blurred disc on funduscopic examinations, requiring further work-ups^11^. It is important to differentiate PHOMS from true papilledema that represents increased intracranial pressure or optic neuritis which can threaten vision loss. However, there exists limited information about PHOMS. Hence, it would be helpful when evaluating the characteristics of patients with PHOMS to understand the condition’s pathogenesis and to confirm the relationship between PHOMS and ODD. This study aimed to evaluate the characteristics of and risk factors associated with PHOMS in children.

## Results

### Baseline characteristics

A total of 112 children, comprising 66 children with PHOMS (PHOMS group) and 46 children without PHOMS (control group) were analyzed in this study. Among the 66 children with PHOMS, 53 (80.3%) had bilateral PHOMS, and 13 (19.7%) had unilateral PHOMS. PHOMS occurred similarly in the right (60) and left (59) eyes. In the PHOMS group, there was no evidence of complications such as disc hemorrhage, nonarteritic anterior ischemic optic neuropathy, retinal vascular occlusion, or choroidal neovascular membrane. None of the patients had concomitant superficial ODD.

Patient demographics and characteristics are listed in Table 1. The mean age of the PHOMS group was 11.7 ± 2.6 (range: 7 to 19) years, while that of the control group was 11.4 ± 3.1 (range: 7 to 19) years. Half of each group was male. All eyes in both groups had best-corrected visual acuity (BCVA) results of 20/25 or greater. In each group, two patients had symptoms of headache (3.0% in PHOMS group and 4.3% in control groups). Mean spherical equivalent (SE) was −3.13 ± 1.87 (range: −8.50 to + 1.00) diopters (D) in the PHOMS group and −0.95 ± 2.65 (range: −6.75 to + 6.00) D in the control group. Additionally, mean astigmatism was 0.67 ± 0.89 (range: 0 to 4.50) D and 0.88 ± 1.02 (range: 0 to 3.50) D in the PHOMS and the control groups, respectively, while mean disc size was 1,735 ± 153 (range: 1,347 to 2,033) µm in the PHOMS group and 1,741 ± 190 (range: 1,296 to 2,404) µm in the control group. Finally, mean ONH tilt angle was 9.84 ± 5.38 (range: 0.00 to 32.09) degrees in the PHOMS group and 3.71 ± 4.41 (range: 0.00 to 15.89) degrees in the control group.

**Table 1:** Demographics and characteristics of peripapillary hyperreflective ovoid mass-like structures (PHOMS) group and control group.

Variables	PHOMS group	Control group	Total	p-value
No. of children/eyes	66/119	46/92	112/211	
Age (years)	11.9 ± 2.7	11.3 ± 3.1	11.6 ± 2.8	0.497
Male:Female	33:33	23:23	56:56	1.000*
Right:Left	60:59	46:46	106:105	1.000*
BCVA ≥ 20/25 (%)	100	100	100	1.000*
Present of headache (%)	3.0	4.3	3.6	1.000*
Mean SE refractive error (diopters)	−3.13 ± 1.87	−0.95 ± 2.65	−2.18 ± 2.48	<0.001
Astigmatism (diopters)	0.67 ± 0.89	0.88 ± 1.02	0.76 ± 0.95	0.121
Disc size (µm)	1735 ± 153	1740 ± 190	1738 ± 170	0.813
ONH tilt angle (degrees)	9.84 ± 5.38	3.71 ± 4.41	7.16 ± 5.83	<0.001

PHOMS = peripapillary hyperreflective ovoid mass-like structures; BCVA = best corrected visual acuity; SE = spherical equivalent; ONH = optic nerve head.

*Fischer’s exact test.

**Figure 1:**
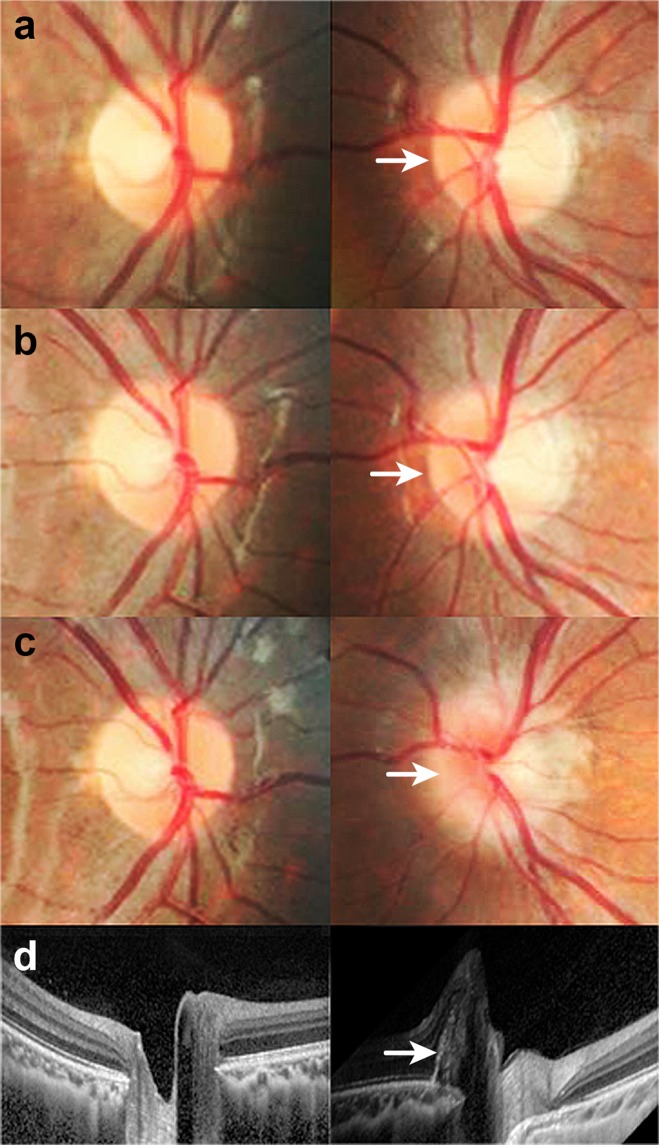
Serial changes (ages 8–11 years) of disc photographs and refractive errors in a male diagnosed with PHOMS. A myopic shift and disc tilt occurred in the left eye with development of PHOMS. (**a**) The optic disc seemed normal in both eyes at the age of eight years. The spherical equivalent (SE) was +1.10 D in the right eye and −0.50 D in the left eye. (**b**) The left disc margin was slightly elevated after 1.5 years. The SE was +1.10 D in the right eye and −1.80 D in the left eye. (**c**) Left disc blurring was aggravated at the age of 11 years, with an SE of +1.10 D in the right eye and −2.50 D in the left eye. (**d**) An EDI OCT image shows PHOMS in the left eye at the age of 11 years.

**Figure 2:**
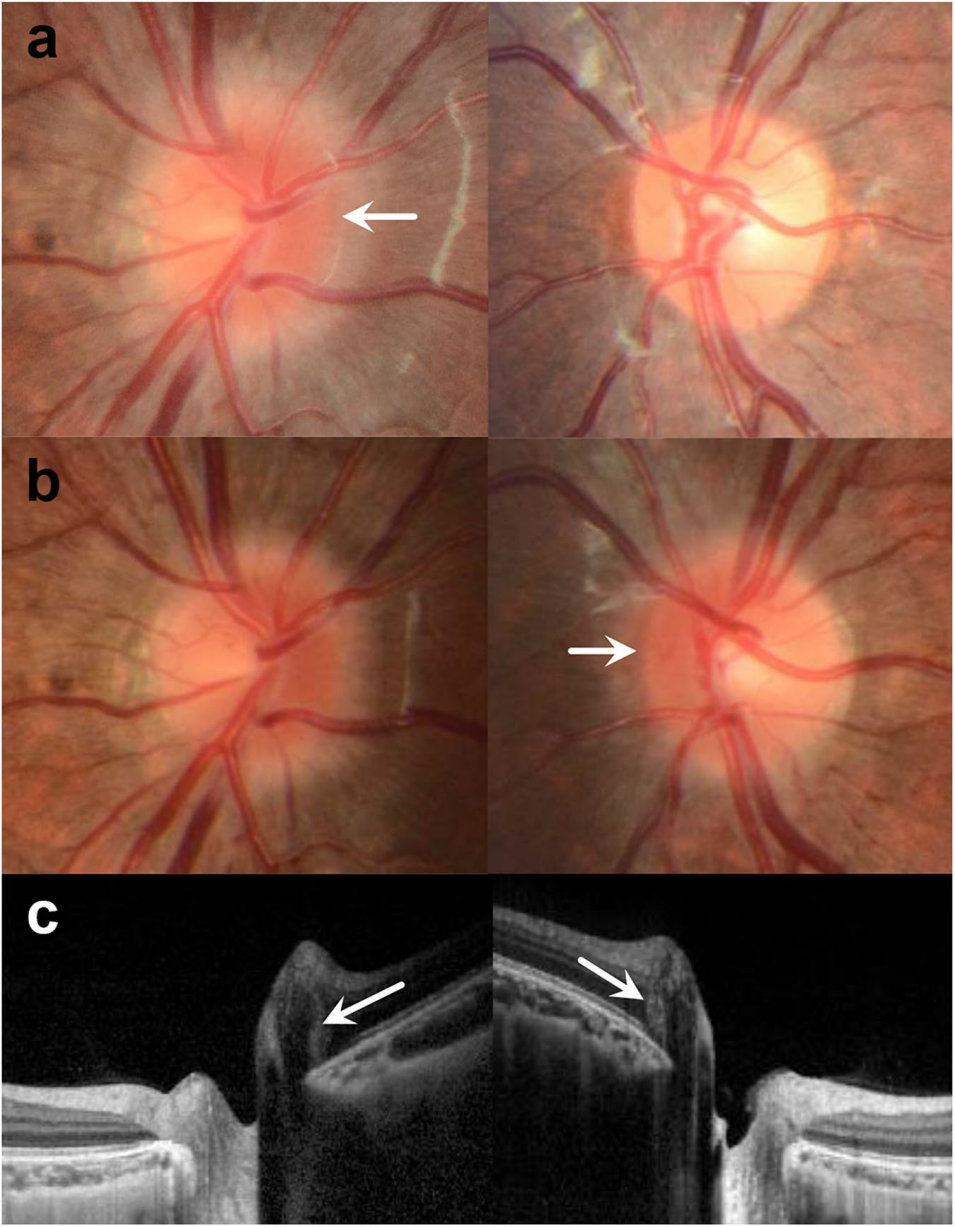
Another example case of serial changes of PHOMS. The patient performed follow-up for orthokeratology lens use in the right eye. (**a**) Disc photographs at the age of nine years, when the SE was −1.75 D in the right eye and −0.25 D in the left eye. The right nasal disc margin was blurred, and the left disc appeared normal. (**b**) At the age of 11 years, the SE was −1.75 D in the right eye and −0.75 D in the left eye. Along with a left myopic shift, new marginal blurring was detected in the left eye. (**c**) An EDI OCT image shows small-sized PHOMS in the right eye.

All eyes with PHOMS presented with myopia of −0.50 D or less, except for one eye with an SE + 1.00 D. In Figs. 1 and 2, we present example cases of PHOMS onset with a concurrent myopic shift.

### Risk factors associated with PHOMS

According to univariable logistic analysis, SE decreased by 1 D [odds ratio (OR): 1.59, 95% confidence interval (CI): 1.35–1.86; *p* < 0.001] and ONH tilt angle increased by one degree (OR: 1.35, 95% CI: 1.24–1.48; *p* < 0.001) in a manner significantly associated with PHOMS. In the multivariable model, decreased SE (OR: 1.71, 95% CI: 1.26–2.32; *p* = 0.001) and increased ONH tilt angle (OR: 1.29, 95% CI: 1.12–1.49; *p* = 0.001) showed a statistically significant association with presence of PHOMS. However, we found no statistically significant difference between the two groups with respect to age, sex, laterality, astigmatism, or disc size (Table 2).

**Table 2:** Risk factors for peripapillary hyperreflective ovoid mass-like structures.

Variables	Univariable model			Multivariable model*		
	OR	95% CI	p-value	OR	95% CI	p-value
Age per 1 year older	1.05	0.92–1.20	0.493			
Male sex	1.00	0.47–2.12	1.000			
Right eye	0.98	0.57–1.70	0.952			
Mean SE refractive error per 1 diopter decrease	1.59	1.35–1.86	<0.001	1.71	1.26–2.32	0.001
Astigmatism per 1 diopter increase	0.80	0.60–1.06	0.124			
Disc size per 100 µm increase	1.00	0.99–1.01	0.812			
ONH tilt angle per 1 degree increase	1.35	1.24–1.48	<0.001	1.29	1.12–1.49	0.001

OR = odds ratio; CI = confidence interval; SE = spherical equivalent; ONH = optic nerve head.

*All variables were adjusted.

### Ganglion cell layer (GCL) changes in PHOMS

We also analyzed GCL thickness in patients with PHOMS via OCT. GCL thickness was slightly decreased in all sectors (temporal, nasal, superior, inferior, and average) in affected eyes compared with in unaffected eyes; however, the difference was not statistically significant in any sector (Table 3).

**Table 3:** Comparison of macular ganglion cell layer thickness in affected eyes and unaffected eyes in patients with peripapillary hyperreflective ovoid mass-like structures.

Variables	Affected eyes (N = 119)	Unaffected eyes (N = 13)	p-value
GCL temporal	49.5	50.4	0.47
GCL superior	53.5	54.0	0.62
GCL nasal	52.2	54.3	0.77
GCL inferior	53.1	53.8	0.32
GCL average	52.1	53.1	0.28

GCL = ganglion cell layer.

### Subgroup analysis of unilateral PHOMS

In a subgroup analysis of 13 patients with unilateral PHOMS, degree of myopia (−3.08 ± 1.77 D and −1.34 ± 2.26 D; *p* = 0.039) and ONH tilt angle (11.40 ± 6.76 and 5.56 ± 6.18 D; *p* = 0.030) were significantly greater in affected eyes than in fellow eyes (Table 4). In all cases of unilateral PHOMS, the affected eyes were more myopic by at least −0.50 D than in the fellow eyes.

**Table 4:** Comparison of affected eyes and fellow eyes in cases with unilateral peripapillary hyperreflective ovoid mass-like structures (PHOMS) (N = 13).

Variables	Affected eyes	Fellow eyes	p-value
Mean SE refractive error (diopters)	−3.08 ± 1.77	−1.34 ± 2.26	**0.039**
Astigmatism (diopters)	0.46 ± 0.71	0.71 ± 0.82	0.446
Disc size (µm)	1696 ± 128	1700 ± 212	0.961
Right:Left	7:6	6:7	1.000*
ONH tilt angle (degrees)	11.40 ± 6.76	5.56 ± 6.18	**0.030**

SE = spherical equivalent; ONH = optic nerve head.

*Fischer’s exact test.

### Correlation between degree of myopia and ONH tilt angle

**Figure 3:**
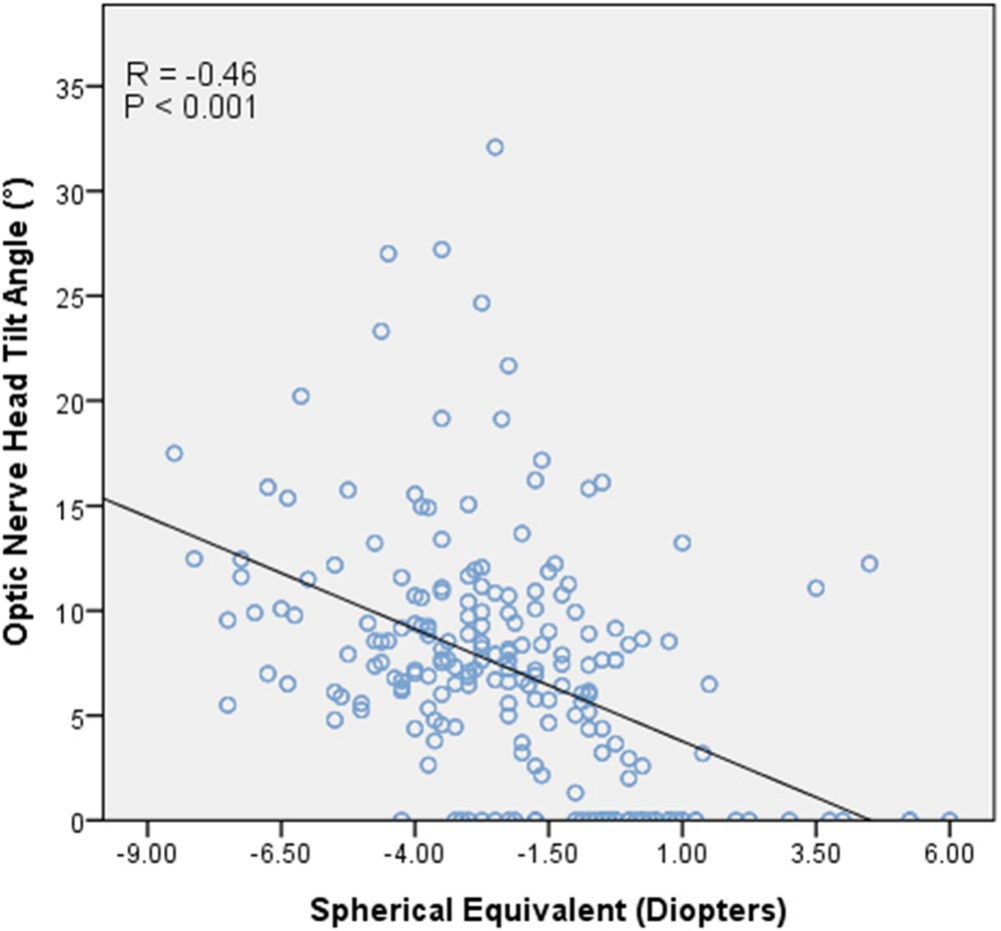
Pearson’s correlation plots showing the correlation between myopia and ONH tilt angle. A significant correlation was observed between SE and ONH tilt angle (*r* = −0.46; *p* < 0.001).

In the correlation analysis between SE and ONH tilt angle, we observed a significant negative correlation (*r* = −0.46; *p* < 0.001) (Fig. 3).

## Discussion

We compared children with PHOMS to controls and found that myopia is a risk factor for PHOMS. In both univariable (OR: 1.59; *p* < 0.001) and multivariable (OR: 1.71; *p* = 0.001) logistic regression analyses, degree of myopia was significantly associated with PHOMS. All eyes with PHOMS had myopia of −0.50 D or less, except for one eye with + 1.00 D of hyperopia. Subgroup analysis of patients with unilateral PHOMS supported this result. The diopters in the affected eyes were smaller than in fellow eyes (−3.08 ± 1.77 D and −1.34 ± 2.26 D, respectively). In addition, ONH tilt angle was another significant risk factor for PHOMS in both univariable (OR: 1.35; *p* < 0.001) and multivariable (OR: 1.29; *p* = 0.001) analysis. Further, we presented several example cases of PHOMS onset along with a myopic shift and ONH tilt, documenting the changes with serial optic disc photographs (Figs. 1 and 2).

Although herniation of distended axons into the peripapillary retina and exoplasmic stasis are suggested pathophysiologic causes of PHOMS^1,10^, the exact pathophysiology remains unknown. We hypothesize that a myopic shift during adolescence is associated with genesis of PHOMS and that optic disc tilt may be a mediator between myopia and PHOMS. Optic disc tilt is a feature of myopic shift, arising from scleral stretching in childhood^12^. Nasal bulging and kinking of retinal nerve fibers might develop in conjunction with the process of disc tilt during myopic shifts. Optic disc tilting also leads to compression of axons and alterations of axonal transport on the nasal side of the ONH, both of which are potential pathogenic causes of PHOMS^9^. We observed a significant correlation between SE and ONH tilt (*r* = −0.46; *p* < 0.001) in this study. Previous studies have also found that axial length or degree of myopia and ONH tilt angle are correlated^13,14^. Our theory is supported by findings of several studies on tilted disc syndrome. Shinohara *et al*. evaluated the morphology of tilted discs in adult patients using swept-source OCT and reported that eyes with tilted disc syndrome showed the protrusion of Bruch’s membrane toward the ONH and herniation of the retinal nerve fiber below the protruded Bruch’s membrane and choroid^15^. Similarly, another study evaluated a novel SD-OCT finding of a dome-shaped hyperreflective structure and its correlation with visual field defects in children with tilted disc syndrome and concluded that the structure in question was consistent with herniated retinal nerve fibers^16^. They speculated that this hyperreflective PHOMS-like lesion is a unique feature of pediatric tilted disc syndrome and may be a result of acute bending of the fibers of oblique insertion of the nerves, leading to a visual field defect. Seo and Park described a case of rapidly progressing PHOMS in a nine-year-old child, which is consistent with the age range in which a myopic shift is usually observed^17^.

Interestingly, we also found several studies presenting the emergence and progression of superficial ODD in childhood. Giuffre reported two cases of ODD and suggested that disc tilt and ODD can have a cause-and-effect relationship due to axonal compression induced by distortion of the scleral canal in tilted discs^18^. Frisén reported a case of ODD that was followed over 23 years. In this case report, drusen showed dynamic morphologic changes from the ages of eight to 16 years, a typical age range of myopia progression^19^. Malmqvist *et al*. followed 8 patients with superficial ODD over 56 years and concluded that progression of ODD occurs before adulthood^20^. This commonality between PHOMS and ODD is probably caused by the shared pathogenesis of compression of axons and axonal stasis.

There are ongoing debates regarding diagnosis of PHOMS vs. buried drusen and whether PHOMS are early ODD^1,10,21^. Further prospective longitudinal studies are needed to better understand PHOMS.

There are several limitations to this study. First, this study was a retrospective cross-sectional investigation. Second, the control group consisted of children who underwent enhanced-depth image (EDI) spectral-domain (SD)-OCT to confirm any ocular anomaly when they showed borderline visual acuity without any underlying disease and improved to the normal range of visual acuity with refractive error correction within three months. Thus, this control groups may not represent a normal population. However, according to the study of a population-based health survey (Korean National Health and Nutrition Examination Survey IV–V), the mean SE of 7,695 Korean participants aged five to 20 years (mean age: 11.8 years) was −1.82 D^22^. Since the Korean National Health and Nutrition Examination Survey evaluated SE by noncycloplegic autorefraction, there may have been some degree of overestimation of myopia in young participants. Therefore, although there were limitations to control group selection in our study, the SE finding of −0.95 ± 2.65 D in the control group using cycloplegic refraction was comparable to that of the normal population. Third, axial length was not evaluated, even though myopia is usually correlated with elongation of ocular axial length. Therefore, further larger prospective longitudinal studies are needed to support our hypothesis and gain a better understanding of PHOMS. Despite these limitations, this is the first reported association between myopia and PHOMS.

According to our findings, myopia increases the risk of PHOMS in children. Disc tilt induced by myopic shifts during childhood may be associated with PHOMS. These findings might increase the understanding of the genesis of PHOMS.

## Methods

This retrospective study was conducted according to the Declaration of Helsinki, after approval by the Institutional Review Board of Nowon Eulji Medical Center.

Children under the age of 20 years who were diagnosed with PHOMS between November 2015 and August 2018 at Eulji University Nowon Eulji Medical Center were reviewed (PHOMS group). The control group consisted of children who underwent EDI SD-OCT to confirm any ocular anomaly when they showed borderline visual acuity and improvement to the normal range of visual acuity with refractive error correction within three months. Children with history of ophthalmologic surgery, neurologic and other ophthalmologic diseases except refractive errors, and systemic diseases were excluded from this study. Informed consent was waived by Institutional Review Board of Nowon Eulji Medical Center, because this study was conducted retrospectively using medical records without identifiable private information and there was no risk to the subjects.

All children underwent a comprehensive ophthalmic examination that included measurement of BCVA, slit-lamp biomicroscopy, cycloplegic refraction, ocular alignment test, dilated fundus examination, and color fundus photography. EDI SD-OCT (Spectralis; Heidelberg Engineering, Dossenheim, Germany) was performed with 24-line radial scan images. All scans were reviewed and evaluated for absence of motion artifacts and good centering on the optic discs. ONH diameters were defined as the Bruch’s membrane opening (BMO) and measured using the built-in measurement tool of the OCT instrument. The mean horizontal and vertical diameters of each plane were used. ONH tilt angle was defined as the angle between the BMO plane and the optic canal plane (i.e., line connecting the nasal BMO and the innermost margin of the externally oblique border tissue)^13^. Images were evaluated using ImageJ version 1.52 (National Institutes of Health, Bethesda, MD, USA) after adjustment to a ratio of 1:1 μm.

We also examined the thickness of the macular GCL in the PHOMS group. Specifically, thickness values of GCL were measured in the Early Treatment Diabetic Retinopathy Study central circular 3-mm-diameter area including the superior, inferior, temporal, and nasal areas^23^. If a patient in the PHOMS group was suspected to have concomitant true disc edema or presented with a condition mimicking papilledema, further follow-up examinations were performed to rule out other causes of disc swelling and to reveal any functional abnormalities. Thus, additional testing may have included: Ishihara color vision test, fluorescein angiography, B-scan, static automated perimetry using a central 30-2 Humphry Field Analyzer (Humphrey Instruments Inc., San Leandro, CA, USA), full-field visual evoked potentials, and brain magnetic resonance imaging. The SE refractive error was calculated as the sphere + 1/2 cylinder as determined by cycloplegic refraction.

### Statistical analysis

Statistical analyses were performed using a commercially available statistical package (SPSS version 23.0 for Windows; IBM Corp., Armonk, NY, USA). Continuous data are presented as mean with standard deviation (SD), and categorical data are presented as counts and percentages. Fisher’s exact test was used to compare categorical data, while the independent *t*-test was used for comparison of continuous parameters. Univariable and multivariable logistic regression analyses were performed to investigate risk factors associated with presence of PHOMS. Correlation between SE and ONH tilt angle was evaluated by Pearson’s correlation analysis and the correlation coefficient (*r*) was calculated. A value of *p* < 0.05 was considered statistically significant.
